# Sizing and phenotyping of cellular vesicles using Nanoparticle Tracking Analysis

**DOI:** 10.1016/j.nano.2011.04.003

**Published:** 2011-12

**Authors:** Rebecca A. Dragovic, Christopher Gardiner, Alexandra S. Brooks, Dionne S. Tannetta, David J.P. Ferguson, Patrick Hole, Bob Carr, Christopher W.G. Redman, Adrian L. Harris, Peter J. Dobson, Paul Harrison, Ian L. Sargent

**Affiliations:** a Nuffield Department of Obstetrics and Gynaecology, University of Oxford, John Radcliffe Hospital, Oxford, United Kingdom; b Nuffield Department of Clinical Laboratory Science, University of Oxford, John Radcliffe Hospital, Oxford, United Kingdom; c NanoSight Ltd., Amesbury, Wiltshire, United Kingdom; d Weatherall Institute for Molecular Medicine, University of Oxford, John Radcliffe Hospital, Oxford, United Kingdom; e Department of Engineering Science, Oxford University Begbroke Science Park, Yarnton, United Kingdom; f Oxford Haemophilia & Thrombosis Centre, Churchill Hospital, Oxford, United Kingdom

**Keywords:** Microvesicles, Microparticles, Exosomes, Nanoparticle Tracking Analysis, Flow Cytometry

## Abstract

Cellular microvesicles and nanovesicles (exosomes) are involved in many disease processes and have major potential as biomarkers. However, developments in this area are constrained by limitations in the technology available for their measurement. Here we report on the use of fluorescence nanoparticle tracking analysis (NTA) to rapidly size and phenotype cellular vesicles. In this system vesicles are visualized by light scattering using a light microscope. A video is taken, and the NTA software tracks the brownian motion of individual vesicles and calculates their size and total concentration. Using human placental vesicles and plasma, we have demonstrated that NTA can measure cellular vesicles as small as ∼50 nm and is far more sensitive than conventional flow cytometry (lower limit ∼300 nm). By combining NTA with fluorescence measurement we have demonstrated that vesicles can be labeled with specific antibody-conjugated quantum dots, allowing their phenotype to be determined.

**From the Clinical Editor:**

The authors of this study utilized fluorescence nanoparticle tracking analysis (NTA) to rapidly size and phenotype cellular vesicles, demonstrating that NTA is far more sensitive than conventional flow cytometry.

## Graphical Abstract

Screen shot from video of light scatter of placental vesicles overlaid with a graph of vesicle size and concentration as determined by nanoparticle tracking analysis.

**Figure:**
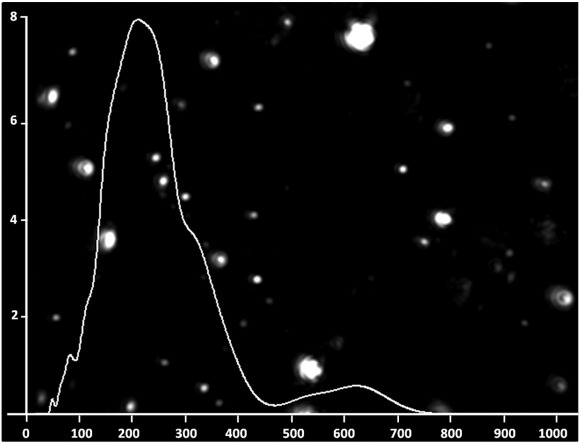

Many cells shed small vesicles in a regulated way; such vesicles have a key role in intercellular communication. In general there are three main types of vesicles: apoptotic bodies (500 nm–3 μm in diameter)^1^ released by cells undergoing apoptosis, microvesicles (100 nm–1 μm), which directly bud from the plasma membrane,^2^ and nanovesicles (30 nm–100 nm), which include exosomes released via exocytosis from multivesicular bodies of the endosome.^3^ All are involved in cell signaling. The properties of cellular vesicles have been reviewed extensively elsewhere,^3-5^ Briefly, (i) they carry diverse membrane and cytosolic proteins as well as messenger and microRNAs; (ii) they can affect the physiology of their target cells in various ways, from inducing intracellular signaling following binding to receptors, to conferring new properties after the acquisition of new receptors, enzymes, or genetic material by fusion or endocytosis^3-5^; (iii) they participate in physiological processes including hemostasis and thrombosis, inflammation, immune interactions, and angiogenesis^4,6^; and (iv) circulating levels are elevated in various disorders, including atherosclerosis and coronary artery disease, pre-eclampsia, hematological and inflammatory diseases, diabetes, and cancer.^4^ They may therefore be useful as prognostic and diagnostic biomarkers for early detection of a wide variety of diseases and have a potential role in monitoring treatment.

Relevant research is constrained by the limitations of current methods of measurement.^7^ Electron microscopy demonstrates the presence of microvesicles and exosomes in ultracentrifuge pellets of biological fluids (e.g., cell culture medium, plasma, or urine) but either is not quantitative and requires extensive sample preparation. The composition of pelleted material can be determined by western blotting, but this gives no information on the number of particles or their sizes. Enzyme-linked immunosorbent assays do not capture all the vesicles present, cannot discriminate between microvesicles and exosomes, and also detect soluble antigens. Conventional flow cytometry, using analogue or first-generation digital instruments, is unable to detect vesicles of <300 nm (which excludes exosomes).^8^ More recently atomic force microscopy has been successfully used to determine the size distribution and number of CD41^+^ vesicles in plasma, but this technique is highly labor intensive.^9^ Dynamic light scattering (DLS), which determines size by light scattered from particles under brownian motion, can measure biological vesicles in suspension. A major disadvantage is that it cannot resolve mixtures of microvesicles and exosomes, because it is biased toward the detection of larger particles.^10^

Here we describe nanoparticle tracking analysis (NTA), a new method for direct, real-time visualization and analysis of nanoparticles in liquids that overcomes many of the problems associated with the above methods.^11^ NTA relates the rate of brownian motion to particle size. In this system vesicles are visualized by light scattering using a light microscope. A video is taken, and the NTA software tracks the brownian motion of individual vesicles and calculates their size and total concentration.

The purpose of this study was (i) to evaluate the potential of NTA for measuring the size and concentration of microvesicles and nanovesicles in biological samples and (ii) to demonstrate, for the first time, how it can be developed to allow the vesicles to be phenotyped using fluorescent antibodies. As mentioned above, cellular microvesicles and nanovesicles can be found in the circulation. However, the vesicles recovered from plasma are derived from many different cell types including platelets, endothelial cells, leukocytes, and red cells, which complicates their analysis. We have therefore initially used vesicles derived from the human placenta as a simple model for developing the techniques described here. The placenta releases microvesicles and nanovesicles into the maternal blood during pregnancy as part of the normal turnover of the placental surface.^12^ Large amounts of highly purified vesicles can be prepared, after delivery, by perfusing the maternal surface of the placenta with buffer, which mimics the maternal blood flow.^13^ The vesicles are then recovered by ultracentrifugation for analysis. We have then demonstrated that NTA can be used to analyze total cellular vesicles in a complex biological fluid, human plasma, using a fluorescent quantum dot-labeled cell tracker peptide.

## Methods

### Preparation of placental vesicles

This study was approved by the Mid and South Buckinghamshire Research Ethics Committee. Placental vesicles were prepared using a dual placental perfusion system.^13^ Placentas were obtained from healthy pregnant women with informed consent at caesarean section, without labor, and were processed immediately. An individual lobule was isolated and the fetal circulation perfused with 0.1-μm filtered modified M-199 tissue culture medium (medium 199 with l-glutamine and Earle's salts, containing 0.8% dextran 20, 0.5% bovine serum albumin, 5000 U/L sodium heparin, and 2.75 g/L sodium bicarbonate, pH 7.4) containing a bolus of 100,000 IU streptokinase to promote clot removal, at a rate of 5 mL/min. The whole placenta was turned upside down and laid inside a Perspex water jacket maintained at 37°C. Maternal perfusion medium (0.1 μm filtered M-199 with l-glutamine and Earle's salts, containing 0.5% bovine serum albumin, 5000 U/L sodium heparin, and 2.75 g/L sodium bicarbonate, pH 7.4) established the maternal circulation through eight 1.7-mm fetal feeding tubes at a controlled rate of 20 mL/min. Perfusion media were warmed in a 37°C water bath, and the maternal perfusion medium was oxygenated with 95% O_2_, 5% CO_2_. The lobule was perfused for 20 minutes to equilibrate the system, after which the maternal circuit containing 600 mL of perfusion medium was closed. The volume of fetal effluent was measured every 2 minutes and the oxygen concentration of the maternal-side perfusate monitored continuously to ensure stability. Pressure monitors were used on both the maternal and fetal sides of the placenta to ensure no significant deviations from baseline occurred during the experimental period. After 3 hours the maternal perfusate was centrifuged in a Beckman J6-M centrifuge (Beckman Coulter, High Wycombe, United Kingdom) at 600 *g* for 10 minutes at 4°C. The remaining supernatant was centrifuged at 150,000 *g* for 1 hour at 4°C in a Beckman L8-80M ultracentrifuge (Beckman Coulter). The resultant pellets were pooled and washed in phosphate buffered saline (PBS) and then resuspended in PBS to a final total protein concentration (BCA protein assay kit; Pierce, Thermo Scientific, Rockford, Illinois) of 5 mg/mL and stored in aliquots at −80°C until use.

### Electron microscopy

For routine electron microscopy, formvar/carbon-coated grids were floated on 50 μL drops of placental vesicle suspension, washed in distilled water, and negatively stained with 1% methyl tungstenate before examination in the electron microscope. For immuno-electron microscopy, formvar/carbon-coated grids were floated on 50 μL drops of placental vesicle suspension. The vesicles were then labeled with the monoclonal antibody NDOG2, specific for placental alkaline phosphatase, which is expressed on placental vesicles.^14^ Grids were washed on drops of PBS and then placed on a drop of primary antibody (NDOG2 1:50) in PBS for 20 minutes, washed in PBS, and placed on a drop of secondary antibody (goat anti-mouse 1:25) conjugated to 10 nm colloidal gold for 20 minutes. The grids were then washed in PBS, fixed with glutaraldehyde, washed in distilled water, and negatively stained with 1% methyl tungstenate before examination in the electron microscope. Vesicle diameters were measured manually from the electron micrographs by two independent observers and the mean vesicle diameter calculated.

### Nanoparticle tracking analysis

**Figure 1:**
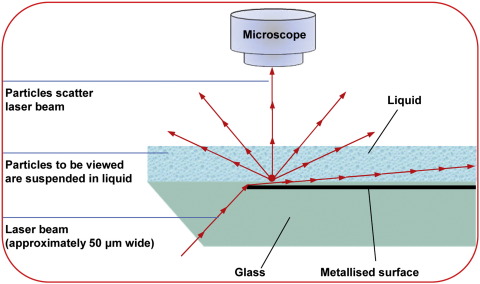
NanoSight instrument configuration.

The system, the NanoSight LM10 (NanoSight Ltd., Amesbury, United Kingdom), uses a finely focused laser beam that is introduced to the sample (a liquid containing a dilute suspension of particles) through a glass prism (Figure 1). The beam refracts at a low angle as it enters the sample, resulting in a thin beam of laser light that illuminates particles through the sample. Particles resident within the beam are visualized using a conventional optical microscope, fitted with a video camera, aligned normally to the beam axis, which collects light scattered from all particles in the field of view. The sample chamber is ∼500 μm deep, but the beam depth is around 20 μm at the point of analysis, matching with the depth of focus of the imaging optics. A video of typically 60 seconds duration is taken, with a frame rate of 30 frames per second, and particle movement is analyzed by NTA software (NanoSight Ltd.). The NTA software is optimized to first identify and then track each particle on a frame-by-frame basis, and its brownian movement tracked and measured frame to frame. The velocity of particle movement is used to calculate particle size by applying the two-dimensional Stokes-Einstein equation:

$$<x,y{>}^{2}=\frac{{\text{K}}_{\text{B}}{\text{T}}_{{\text{t}}_{\text{s}}}}{3\pi \eta d_{\text{h}}}$$

where <x,y>^2^ is the mean squared displacement, K_B_ is Boltzmann's constant, T is the temperature of the solvent in Kelvin, t_s_ is the sampling time (i.e., 1/30 fpsec = 33 msec), η is the viscosity, and d_h_ is the hydrodynamic diameter.

A range of parameters can be adjusted both in video capture (such as camera gain and shutter speed) and in analysis (such as filter settings, background subtraction, minimum required track length, and frame-to-frame search area), thus allowing the user to optimize particle identification and tracking for a particular sample. The range of sizes that can be analyzed by NTA depends on the particle type—that is, whether it has a high (e.g., colloidal gold) or a low refractive index (e.g., cellular vesicles). The lower limit is a function of the signal-to-noise ratio of the image and is thus strongly affected by the amount of light scattered. The particles in consideration here (i.e., at the limit of detection of the system) are in the Rayleigh scattering regime. In this regime the amount of light scattering is given by:

$$\sigma_{s}=\frac{2\pi^{5}}{3}\frac{d^{6}}{\lambda^{4}}{\left(\frac{n^{2}-1}{n^{2}+2}\right)}^{2}$$

where *d* is the particle diameter, λ is the wavelength of light, and *n* is the ratio of the particle refractive index to the solvent refractive index. Generally speaking, cellular vesicles have a low refractive index, and the smallest detectable size using the NTA system is in the order of 50 nm. At ∼1 μm the brownian motion of a particle becomes too limited to track accurately.

To demonstrate the ability of NTA to discriminate particles of different sizes in a polydisperse sample, a 5:1 mixture of 100 nm and 300 nm polystyrene beads (NIST beads; Thermo Scientific, Fremont, California) was analyzed. To demonstrate how the particle concentration can be estimated, 100 nm beads at a range of concentrations between 2 × 10^8^ and 20 × 10^8^ particles per milliliter were analyzed. Placental vesicles, stored in PBS at −80°C, were thawed at room temperature (18−25°C) before NTA analysis. Each sample was diluted in PBS over a range of concentrations between 2 × 10^8^ and 8 × 10^8^ vesicles per milliliter. The samples were mixed before introduction into the sample chamber and a video recording, typically 1 minute, initiated. A combination of high shutter speed and gain followed by manual focusing enables optimum visualization of a maximum number of vesicles. To accurately track the vesicles they must be visualized as single points of light. The NTA software is unable to effectively track very large vesicles or those with confounding Newton rings. In this case the shutter speed and gain are reduced accordingly (see Supplementary Videos S1-S3, which can be found in the online version of this article). For highly polydisperse samples, multiple analyses at a range of settings may be necessary. The samples were advanced between each recording to perform replicate measurements. NTA post-acquistion settings were optimized and kept constant between samples, and each video was then analyzed to give the mean, mode, and median vesicle size together with an estimate of the concentration. An Excel spreadsheet (Microsoft Corp., Redmond, Washington) was also automatically generated, showing the concentration at each vesicle size.

### Immunofluorescence labeling with NDOG2 antibody conjugated to quantum dots

Quantum dots were conjugated to NDOG2 antibody with a Qdot 605 Antibody Conjugation Kit (Invitrogen, Paisley, United Kingdom) coated with polyethylene glycol amine (molecular weight 2000) according to the manufacturer's instructions. Briefly, quantum dots were activated with the cross-linker 4-(maleimidomethyl)-1-cyclohexanecarboxylic acid *N*-hydroxysuccinimide ester (SMCC), yielding a maleimide-nanocrystal surface. Excess SMCC was removed by size exclusion chromatography. The antibody was then reduced by dithiothreitol to expose free sulfhydryl groups, and excess dithiothreitol was removed by size exclusion chromatography. The activated quantum dots were covalently coupled with reduced antibody and the reaction quenched with mercaptoethanol. Conjugates were concentrated by ultrafiltration and purified by size exclusion chromatography. Mouse immunoglobulin G was labeled in the same way for use as the isotype control. Placental vesicle suspension (see above) was diluted 1:100 and NDOG2-conjugated quantum dots were added to give a final quantum dot concentration of 10 nM. The molar ratio of antibody to quantum dots was 3.5:1. After a 15 minute incubation at room temperature, the labeled vesicles were diluted in PBS for analysis by NTA.

### Flow cytometry

Placental vesicles were analyzed using a Becton Dickinson LSRII flow cytometer (Becton Dickinson, Oxford, United Kingdom) equipped with a 405 nm (violet) and 488 nm (blue) laser. A vesicle gate was determined using calibration microspheres of various sizes (200 nm, 290 nm, 390 nm, 590 nm; Duke Scientific Corporation, Palo Alto, California) and 1 μm Fluoresbrite Plain YG beads (Polysciences Inc, Warrington, Pennsylvania). Using a side-scatter (SSC) threshold of 200 arbitrary units the lower sensitivity of the instrument was established and the SSC and forward scatter (FSC) voltages were set. There is overlap in the 200 nm bead population and the instrument noise (determined by running PBS filtered through a 100 nm filter). Therefore, a gate was set to include vesicles of 300 nm-1 μm in diameter. Vesicles were enumerated by running Becton Dickinson TruCount Tubes (three tubes per experiment) to establish the flow rate of the system. All samples were run on the “LO” setting with a flow rate of 12 μL per minute. Placental vesicles were diluted 1:50 in PBS and incubated with 10 μL of FcR receptor block at 4°C for 10 minutes. They were then incubated with NDOG2-Qdot605 or IgG_1_-Qdot605 at 10 μg/mL and incubated for 15 minutes at room temperature. Samples were then diluted to 1 mL with PBS and analyzed by flow cytometry.

### Fluorescence NTA

The NanoSight NS500 instrument (NanoSight Ltd.) is a development of the NanoSight LM10 that allows the detection of microvesicles and nanovesicles labeled with stable fluorophores. It uses a 405 nm (violet) laser diode to excite suitable fluorophores whose fluorescence can then be determined using a matched 430 nm long-pass filter. Measurements are made in fluorescence mode with the long-pass filter in place so that only fluorescently emitted light is measured. The fluorescent particles are individually tracked in real time, from which labeled particle size and concentration can be determined. Under light scatter mode, the total number of particles can be measured and subsequently compared to the concentration of labeled particles. The calibration beads used were Nanosphere Size Standards 200 nm [1.7% coefficient of variation (CV); Thermo Scientific] and Fluoresbrite Plain YG 100 nm Microspheres (5.8% CV) (Polysciences Inc.).

#### Labeling of plasma cellular vesicles with fluorescent QTracker dye

Platelet-poor plasma was prepared from blood collected into 0.106 mM trisodium citrate, by double centrifugation at 2500 *g* for 15 minutes. Cellular vesicles were either labeled directly in the plasma or after ultracentifugation of platelet-poor plasma at 100,000 *g* for 1 hour at 4°C. Vesicles were labeled using the QTracker cell labelling kit (Invitrogen, Carlsbad, California), which uses a targeting peptide to deliver QDot nanocrystals across the plasma membrane into the cytoplasm.^15^ Briefly, 2 μL of a 0.625 nM solution of QTracker quantum dots were added to 200 μL of plasma or plasma vesicle pellet resuspended in PBS and incubated at 37°C for 1 hour. NTA analysis was performed using the NanoSight NS500 instrument as described above.

## Results

### NTA measurement of particle size and concentration

**Figure 2:**
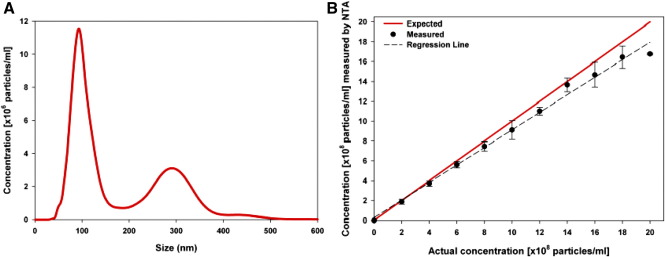
NTA measurement of particle size and concentration. **(A)** Mixture of 100 nm and 300 nm polystyrene beads (ratio 5:1) analyzed by NTA. **(B)** NTA analysis of 100-nm beads at a range of concentrations from 2 × 10^8^ to 20 × 10^8^ per milliliter, mean ± SD of five replicates. The actual values for the concentration of the beads are shown on the *x*-axis and those measured by NTA on the *y*-axis.

Figure 2, *A* shows that NTA successfully addresses one of the key problems with DLS (i.e., polydispersity), in that it can resolve and accurately measure different-size particles (here 100 nm and 300 nm polystyrene beads) simultaneously within the same solution. Although the distributions partly overlap, the two peaks of each bead subtype at 100 nm and 300 nm peaks are clearly discriminated. This is an essential prerequisite for analyzing cellular microvesicles and nanovesicles in biological fluids. The overlap of the peaks in Figure 2, *A* is due to the inherent limitation of measuring a stochastic process (brownian motion) by sampling over a finite time period (the time for which each particle can be tracked). For analysis of beads where the particle size distribution is well defined, the NTA software can correct for this and produce tighter peaks. However, for analyzing cellular vesicles where we cannot make assumptions about the size distribution, we chose not to use this correction, resulting in the apparent poorer resolution seen here.

NTA also allows the particle concentration to be estimated directly, with good linearity being seen between the actual concentration and that measured by NTA in the range of 1 × 10^8^-8 × 10^8^ beads per milliliter for monodisperse 100 nm beads (Figure 2, *B*). Concentration measurement is less precise in polydisperse samples. This is due to the need to use instrument settings (camera shutter speed and gain) that are optimal for smaller (e.g., 100 nm) particles to ensure that they are included in the analysis. However, this leads to an overestimate of the concentration of the larger particles (e.g., 300 nm), because at these settings they may scatter multiple points of light, which are interpreted by the software as individual particles (see Supplementary Video S2).

### Comparison of NTA, electron microscopy, and flow cytometry analysis of placental vesicles

**Figure 3:**
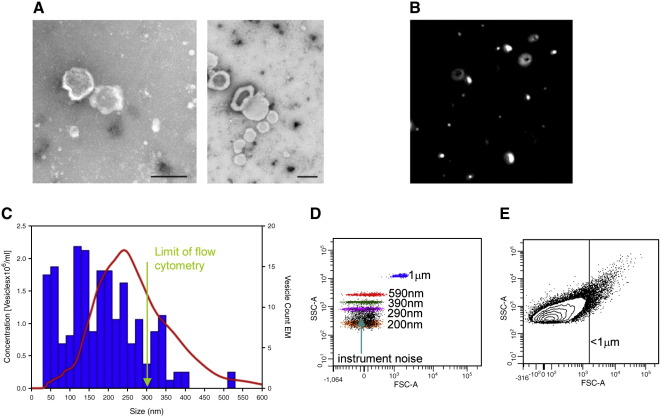
Comparison of NTA, electron microscopy (EM), and flow cytometry analysis of placental vesicles. **(A)** Electron micrograph of placental vesicle ultracentrifuge pellet. Scale bars, 200 nm. **(B)** Screen shot of video from NanoSight LM10 showing optimal light scatter from placental vesicles. **(C)** Vesicle size determined by direct measurement from electron micrographs (blue bars) and NTA (red line). **(D)** Size resolution of marker beads by forward (FSC) and side scatter (SSC) in flow cytometry. Instrument noise was defined by running 100-nm-filtered PBS. **(E)** Placental vesicle FSC and SSC by flow cytometry.

The size distribution of placental vesicles was determined by measuring their diameters directly from electron micrographs of ultracentrifuge pellets. This showed a polydisperse sample with vesicles ranging in size from 20 nm to 600 nm with a peak size between 100 and 140 nm (Figure 3, *A*). Figure 3, *B* is a screenshot of the same sample analysed on the NanoSight LM10 showing a range of vesicle sizes. NTA gave a similar vesicle size distribution from 40 to 600 nm with a peak around 250 nm (Figure 3, *C*). The peak of smaller material (20−60 nm) seen by electron microscopy but not by NTA is partly due to the lower sensitivity of the LM10 instrument in this size range, but shrinkage artifacts during fixation for electron microscopy can also lead to undersizing of the vesicles.^16,17^

We then demonstrated that NTA can measure vesicles that cannot be detected by flow cytometry. Figure 3, *D* shows a series of beads of standard sizes, analyzed by flow cytometry on a FSC (size) versus SSC (granularity) plot. On our instrument (Becton Dickinson LSRII), FSC can resolve beads of 1 μm from 590 nm diameter, but nothing smaller. By adding the SSC measurement, 200 nm beads can be detected but appear in the same region as instrument “noise.” On this basis, a 300 nm cutoff is a more realistic limit for practical use. The placental vesicle preparation was then analyzed on these settings by flow cytometry (Figure 3, *E*). The majority (>90%) of the vesicles were below 1 μm in diameter, with the main population at around 300-400 nm, cut off to the 300 nm detection limit. The vesicle count was 1.6 × 10^9^ per milliliter. When the same sample was analyzed by NTA, ∼70% of the vesicles were <300 nm and the vesicle count was 3.7 × 10^11^ per milliliter. These results demonstrate that NTA can measure the size and estimate the concentration of biological microvesicles and nanovesicles as small as ∼50 nm, with a sensitivity much greater than flow cytometry.

### Fluorescence NTA of placental vesicles

A limitation of conventional NTA is that although it can accurately measure vesicle size and concentration, it cannot phenotype vesicles or determine their cell of origin in the way that flow cytometry can. The NanoSight instrument has therefore been developed to incorporate a 405-nm blue-violet laser and more sensitive camera to detect fluorescent particles (NanoSight NS500). Fluorophores (quantum dots) attached to antibodies or other biological probes are excited; by using a 430-nm long-pass fluorescence filter, only vesicles that are fluorescently labeled are tracked.

**Figure 4:**
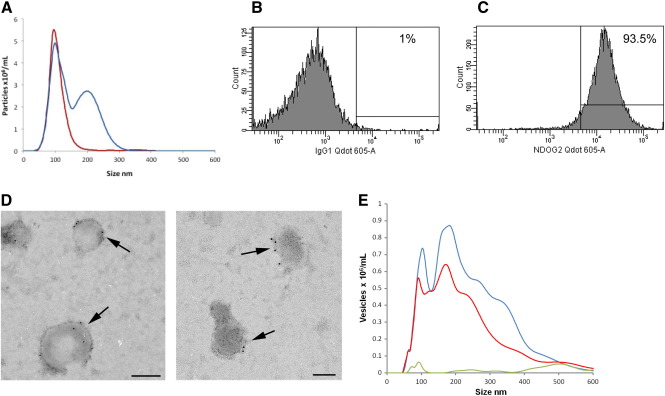
Fluorescence NTA analysis of placental vesicles. **(A)** Mixture of fluorescent 100 nm and nonfluorescent 200 nm beads analyzed on the NanoSight NS500 using light scatter from the 405 nm violet laser (blue line) and the same bead mixture analyzed for fluorescence only using a 430 nm filter (red line). **(B)** Placental vesicles labeled with mouse IgG1 isotype control antibody conjugated to quantum dots and analyzed by flow cytometry. **(C)** Placental vesicles labeled with antibody NDOG2 specific to placental vesicles conjugated to quantum dots and analyzed by flow cytometry. **(D)** Immunogold labeling of placental vesicles with NDOG2 antibody. **(E)** Placental vesicles labeled with antibody NDOG2 conjugated to quantum dots and analyzed on the NanoSight NS500 in light scatter (blue line) and fluorescence (red line) modes. The green line shows placental vesicles labeled with the mouse IgG1 quantum dot isotype control antibody analyzed in fluorescence mode.

To demonstrate the principle, we first analyzed a mixture of 100-nm fluorescent beads and 200 nm nonfluorescent beads. Figure 4, *A* shows the results using NTA in light scatter mode (blue line). Although the distributions partly overlap for the reasons discussed above, the two peaks of each bead subtype at 100-nm and 200 nm peaks are clearly discriminated. However, with the fluorescence filter in place only the fluorescent 100 nm beads are tracked (red line). The size and concentration of 100-nm fluorescent beads, either in light scatter or in fluorescence mode, gave very similar results. We then conjugated quantum dots to a mouse monoclonal IgG1 antibody (NDOG2), which is specific for placental vesicles,^14^ and confirmed that the conjugation was successful by showing that 93.5% of the vesicles detectable by flow cytometry had become labeled with the antibody (Figure 4, *C*), compared to the quantum dot-labeled IgG1 isotype control (Figure 4, *B*). We confirmed that the NDOG2 antibody bound to placental vesicles smaller than 300 nm by immunogold electron microscopy (Figure 4, *D*). The same samples were then analyzed on the NanoSight NS500 instrument, first on light scatter and then in fluorescence mode. Figure 4, *E* shows the NTA of light scatter and fluorescence (see Supplementary Video S4) for vesicles labeled with NDOG2-quantum dots and IgG-quantum dot control. The size distributions of the NDOG2-labeled vesicles are similar, with peaks in the region of 100 nm and 180 nm (range 50−600 nm) whether measured in light scatter or fluorescence mode. The small differences between the peak sizes in light scatter and fluorescence modes were within the observed level of variability (CV 8%) for this technique. The concentration of NDOG2-positive vesicles is slightly lower than that detected by light scatter, possibly as a result of the presence of vesicles from contaminating blood cells in the preparation that will not label with the NDOG2 antibody. The IgG-quantum dot control shows that the NDOG2 antibody labeling of the vesicles is specific (see Supplementary Video S5).

**Figure 5:**
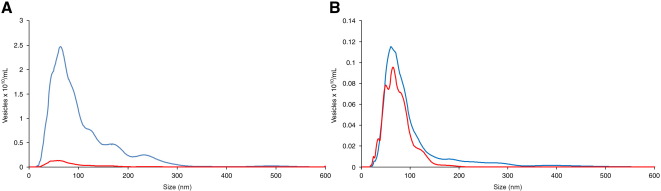
Fluorescence analysis of cellular vesicles in plasma. **(A)** Platelet-free plasma labeled directly with the QTracker cell-labeling reagent coupled to quantum dots and analyzed on the NanoSight NS500 in light scatter (blue line) and fluorescence (red line) modes. **(B)** Ultracentrifuge pellet of the same plasma sample labeled with QTracker and analyzed on the NanoSight NS500 in light scatter (blue line) and fluorescence (red line) modes.

Finally, we analyzed cellular vesicles in human plasma by labeling them with a cell tracker peptide conjugated to quantum dots. Figure 5, *A* shows NTA analysis of platelet-poor plasma labeled with the cell tracker dye in light scatter (blue line) and fluorescence (red line) modes using the NanoSight NS500 instrument. The vesicle size ranged from 50 nm to 300 nm with a peak size around 80 nm. The total vesicle count was 1.49 × 10^12^ per milliliter, whereas the labeled vesicle count was 1.2 × 10^10^ per milliliter. This difference is probably due to the presence of large numbers of lipid vesicles (chylomicrons and very-low-density lipoproteins) in the plasma, which are of a similar size to cellular vesicles^18,19^ and can be detected by light scatter, but do not label with the cell tracker dye. This is supported by the analysis of the vesicles in the plasma ultracentrifuge pellet (Figure 5, *B*), showing very similar counts whether the vesicles were analyzed in light scatter (blue line, 1.4 × 10^10^ per milliliter) or fluorescence (red line, 1.1 × 10^10^ per millliliter) modes. The lipid vesicles would not be pelleted by ultracentrifugation, whereas most of the cellular vesicles are recovered.

## Discussion

Cellular microvesicles and nanovesicles are potential markers of human disease. To use these vesicles in diagnostic tests, a simple and rapid method of simultaneously determining their size, concentration, and phenotype in biological fluids such as plasma and urine is required. This cannot be achieved by existing methods. Our results demonstrate the feasibility of using NTA for this purpose. This technology, although relatively new, is well established in other fields including the measurement of engineered nanoparticles, carbon nanotubes, inks and pigments, protein aggregates, and viral particles.^11^

We have shown that NTA can be used to determine the size and concentration of cellular vesicles and in doing so offers distinct advantages over other methods. NTA is more rapid than electron microscopy and atomic force microscopy, allowing more vesicles to be analyzed and, because the vesicles are analyzed in suspension, they are not subject to shrinkage artifacts due to fixation. Crucially, it can detect nanovesicles much smaller than those that can be seen by conventional flow cytometry (∼300 nm). Although the latest generation of digital flow cytometers can detect beads as small as 100 nm and can discriminate these from 300 nm beads in a mixture,^20^ how these measurements relate to the minimum size of cellular vesicles that can be resolved must be interpreted with caution. Cellular vesicles have a lower refractive index compared to latex or polystyrene beads, which could lead to an underestimation of their size.^20,21^ In contrast, because NTA determines particle size from brownian motion, it is independent of the refractive index of the particle.^11^ Flow cytometry analysis of placental vesicles, which are likely to be composed of a range of exosomes, microvesicles, and apoptotic bodies,^1^ showed that more than 90% were smaller than 1 μm, with the bulk of the population closer to the 300 nm cutoff. NTA analysis confirmed the presence of larger vesicles of 500−600 nm, but these were relatively rare compared to the major population below 300 nm. Although NTA can analyze particles as large as 1 μm in diameter (above this the brownian motion is too slow to measure), the large number of small vesicles in these preparations requires that the sample be diluted for analysis The effect of this is that the number of large vesicles analyzed is significantly reduced, which means their concentration will be underestimated. Thus, studies of large vesicles (>500 nm) alone (e.g., apoptotic bodies) may be better carried out by flow cytometry than by NTA.

DLS can also detect nanovesicles, but it cannot accurately resolve heterogeneous mixtures of vesicles.^22^ This is because a single detection element collects light from all particles simultaneously; meaning that the estimate of particle size and size distribution is biased to a larger particle size. In contrast, NTA simultaneously measures particle size and scattering intensity on individual particles, thus allowing heterogeneous particle mixtures to be resolved. Furthermore, the ability of NTA to see particles directly and individually allow the particle concentration to be estimated from extrapolating the number of particles seen at any given instant to a particle concentration per unit volume through knowing the scattering volume. This facility, which is unobtainable by conventional DLS methods, is invaluable for studying particles in biological fluids, because it allows the measurement of changes in the concentration of particles of different sizes between normal and disease states. Thus, the NanoSight instrument allows a quantitative estimation of sample size, size distribution, and concentration.

The disadvantage of NTA in its original form was that it was unable to determine the phenotype of the vesicles. Biological fluids such as plasma and urine will inevitably contain mixtures of vesicles derived from many different cell types. It is therefore crucial to be able to determine the cellular origin of the vesicles and, to understand their biological function, the molecules that they express on their surface. Here we have successfully adapted and developed this technology by adding a fluorescence capability and demonstrated the proof of principle by measuring placental vesicles labeled with a specific antibody conjugated to quantum dots and cellular vesicles in human plasma using a quantum dot-labeled fluorescent cell tracker peptide. Labeling with the cell tracker peptide clearly shows that a large proportion of the vesicles in plasma are not derived from cells but are probably lipid vesicles, because they were removed by ultracentrifugation and the cellular vesicles pelleted. Experiments are now under way to label plasma vesicles with a panel of antibodies to platelet, erythrocyte, leukocyte, and endothelial cell markers to determine their composition. In summary, NTA in effect extends the power of flow cytometry downward by nearly one order of magnitude in terms of particle size and opens up new possibilities for research into microvesicles and nanovesicles.

## Appendix A Supplementary data

**Use of multiple acquisition settings to analyse different particle sizes by NTA**

Video 1 100nm polystyrene beads analysed at high gain and shutter speed. The beads are visualised as single points of light, which is ideal for sizing and quantitation.

Video 2 A mixture of 100nm and 300nm polystyrene beads analysed at high gain and shutter speed. The 100nm beads are visualised as single points of light, allowing sizing and quantitation. 300nm beads appear as larger over-exposed objects with Newton's rings, which are more difficult to track by NTA which ideally requires the particles to be visualised as a single point of light.

Video 3 The same mixture of 100nm and 300nm polystyrene beads analysed at low gain and shutter speed. The exposure of the 300nm beads is now more suitable for analysis of the larger particles.

**Measurement of fluorescent antibody labelled vesicles**

Video 4 Placental vesicles labelled with anti-placental antibody NDOG2-quantum dots and analysed with the fluorescence filter in place.

Video 5 Placental vesicles labelled IgG-quantum dot control and analysed with the fluorescence filter in place.
