# Supplementary figures and images for: Sizing and phenotyping of cellular vesicles using Nanoparticle Tracking Analysis
Source: Nanomedicine. 2011 Dec;7(6):780–8. doi: 10.1016/j.nano.2011.04.003 (PMC3280380; doi:10.1016/j.nano.2011.04.003)

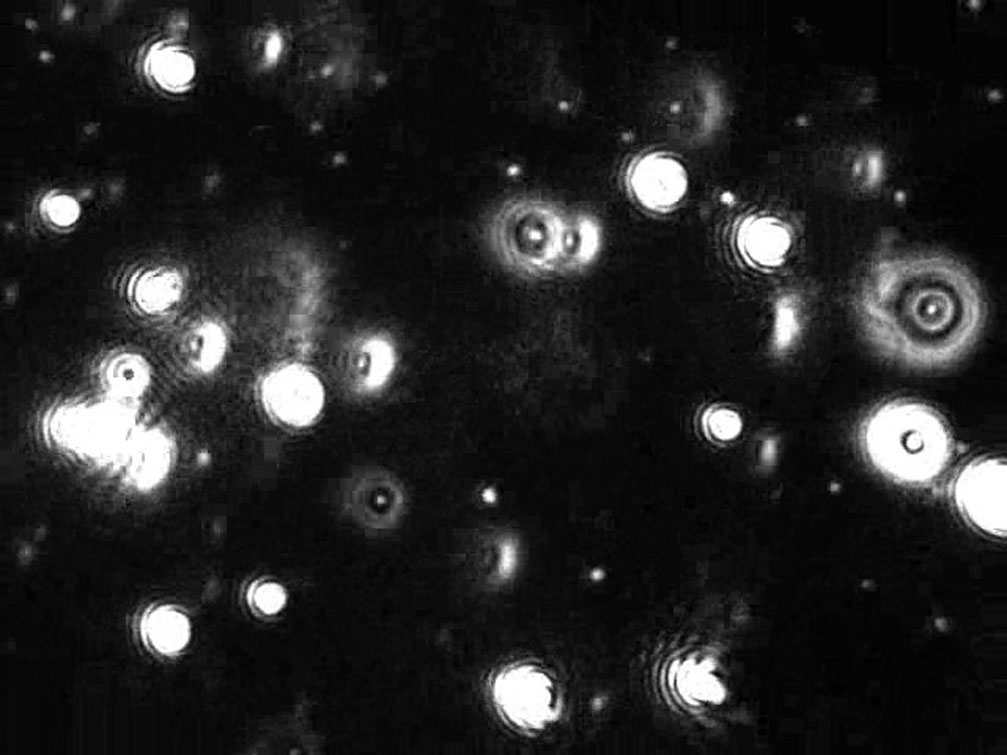

Supplement: Video 2 — A mixture of 100nm and 300nm polystyrene beads analysed at high gain and shutter speed. The 100nm beads are visualised as single points of light, allowing sizing and quantitation. 300nm beads appear as larger over-exposed objects with Newton's rings, which are more difficult to track by NTA which ideally requires the particles to be visualised as a single point of light. [file mmc2.jpg]

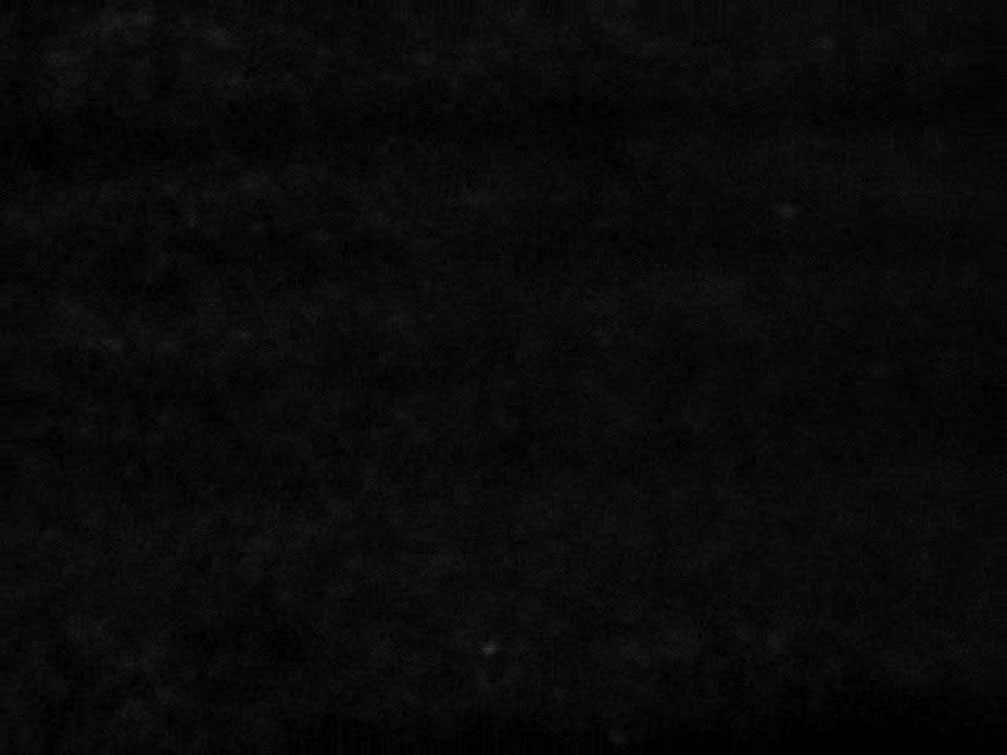

Supplement: Video 5 — Placental vesicles labelled IgG-quantum dot control and analysed with the fluorescence filter in place. [file mmc5.jpg]
